# How Can We Better Assist Caregivers With Understanding and Addressing the Cognitive Health Needs of People With Psychotic Disorders?

**DOI:** 10.1093/schbul/sbaf062

**Published:** 2025-05-22

**Authors:** Shayden Bryce, Amanda Sorenson, Debbie Warner, Alex Stainton, Alice Medalia, Caroline Cellard, Isabel Zbukvic, Jacquie Uren, Jessy Smith, Lauren Libeson, Wilma Peters, Kelly Allott

**Affiliations:** Orygen, Parkville, Victoria, Australia; Centre for Youth Mental Health, University of Melbourne, Parkville, Victoria, Australia; Alfred Mental and Addiction Health, Melbourne, Victoria, Australia; Orygen, Parkville, Victoria, Australia; Haileybury, Keysborough, Victoria, Australia; Alfred Mental and Addiction Health, Melbourne, Victoria, Australia; Orygen, Parkville, Victoria, Australia; Centre for Youth Mental Health, University of Melbourne, Parkville, Victoria, Australia; New York State Psychiatric Institute, Department of Psychiatry, Columbia University Vagelos College of Physicians and Surgeons, New York, United States; Ecole de Psychologie, Université Laval, Québec, Canada; Orygen, Parkville, Victoria, Australia; Centre for Youth Mental Health, University of Melbourne, Parkville, Victoria, Australia; Alfred Mental and Addiction Health, Melbourne, Victoria, Australia; Orygen, Parkville, Victoria, Australia; Alfred Mental and Addiction Health, Melbourne, Victoria, Australia; Orygen, Parkville, Victoria, Australia; Centre for Youth Mental Health, University of Melbourne, Parkville, Victoria, Australia; Orygen, Parkville, Victoria, Australia; Centre for Youth Mental Health, University of Melbourne, Parkville, Victoria, Australia

**Keywords:** schizophrenia, caregiver, family, cognition, education, treatment

## Abstract

Cognitive impairment is a prominent feature of psychosis-spectrum disorders that impedes functional recovery. Globally, clinical guidelines recommend that evidence-based treatments, including cognitive remediation and cognitive compensation, are offered to people with psychosis with cognitive impairment. Clinical guidelines also recommend that, where possible, family is involved in the mental health treatment of their loved ones more broadly. Nevertheless, there is little guidance on how to assist family members with understanding and addressing the cognitive health needs of people with psychotic disorders. This is despite a demonstrable relationship between this symptom domain and caregiver burden as well as a clear need for greater professional supports from the perspectives of consumers and carers. In this article, we highlight the impact of cognitive impairment in psychosis on caregiver outcomes and argue the need to increase efforts to promote knowledge about cognitive health among caregivers via cognition-specific psychoeducation and/or more active involvement in cognitive rehabilitation. We showcase some of the existing cognition-specific resources that are available to caregivers and propose areas in need of future research. We conclude this article by presenting several practical recommendations for how clinical teams can advance their support of family members caring for loved ones with psychosis and cognitive impairment when it is clinically appropriate to do so and the consumer their caregiving network are agreeable.

Cognitive impairment is a prominent feature of psychosis-spectrum disorders. It is highly prevalent, persists during periods of clinical symptom remission, and impedes functional recovery (eg, disrupts efforts to return to education or work). Evidence-based treatments, such as cognitive remediation or compensation, are recommended for people with psychotic disorders given the cognitive and functional benefits of these interventions.^1–5^ There is a burgeoning focus on direct consumer involvement in the development, implementation, and evaluation of cognitive treatments in psychosis, with an increasingly accepted viewpoint that meaningful treatment innovation may only truly occur when working in partnership with consumers and their support network.^6,7^ Nevertheless, little is known about the extent to which family is currently involved, or should be involved, in efforts to promote cognitive health in psychotic disorders. This is despite knowledge that family collaboration is often critical for maintaining healthcare engagement among people with psychosis, and family members have a supporting role in unifying the treatment goals and expectations of consumers and clinical teams.^8,9^ The goal of this article is to highlight the importance of educating support-seeking family members about cognitive impairment and the ways that they can be supported to promote their loved one’s cognitive health. We use the terms “family” and “caregivers” interchangeably. We also use the term “psychosis” to include all psychotic disorders, but acknowledge the variability in relation to the degree of cognitive impairment and effect of cognitive treatments across disorders, with most of the evidence advanced in schizophrenia.

The provision of support to family members caring for people with psychotic disorders and cognitive impairment is a clinical priority according to both consumers and caregivers.^10–12^ Many people live in the family home during their illness^13–15^ and want family involved in their care to help them cope with clinical symptoms, offer support with functional tasks, and/or ease caregiver’s distress.^16–18^ Similarly, caregivers—who are most commonly the parents of a person with a psychotic disorder—report that cognitive impairments affect their loved one’s functioning and are exceedingly difficult to manage,^10,19^ contributing to psychosocial, financial, and functional facets of caregiver burden.^20,21^ Family members may also perceive a large degree of responsibility to help drive their loved one’s functional recovery such as teaching them management strategies, despite not always having the practical or psychosocial resources to do this effectively.^21,22^ This additional responsibility may further contribute to caregiver distress or compound adverse caregiver outcomes, including fewer vocational opportunities, social isolation, emotional exhaustion, or negative caregiving appraisals or changes in identity, especially if family members are already spending large amounts of time providing direct support to loved ones.^15,23–26^

The involvement of family in the treatment and management of cognitive health in psychosis is clearly important given the prevalence, consumer preferences, and wide-ranging impact of cognitive impairments in psychosis. Clinical practice guidelines for the treatment of psychosis recommend that family members receive psychoeducation about the illness and are supported with adaptive coping strategies for themselves and the consumer.^2^ However, while some cognitive health resources exist for families, there is a need for more globally accessible, culturally sensitive guidance on the best ways to support caregivers navigate the *cognitive symptoms* of psychosis specifically.^27^ Recent literature indicates that families value educational resources and primarily access this information online (87%) or directly from a healthcare professional (41%).^8^ Nevertheless, they also want more information from clinical teams about specific symptoms in psychotic disorders (eg, negative/cognitive symptoms), the types of available treatments, opportunities for skill development, and ways that they can support their loved one to function independently with greater confidence.^8,28^ Routine access to this type of information in relation to cognitive impairment does not appear to be common practice for families supporting people with psychotic disorders.

## Caregivers Need Information About Cognitive Impairment in Psychosis

Caregivers providing support to family members with complex illnesses including psychosis describe a critical need for information, skill development, and professional support.^28,29^ Thus, like many other forms of treatment, providing psychoeducation about cognitive functioning in psychosis is a foundational component of involving them in the management of cognitive impairment. People with psychosis report that cognitive problems are distressing but struggle to separate these from clinical symptoms or describe their challenges on a day-to-day level.^30^ Similarly, family members observing the deterioration of their loved one may have had minimal exposure to cognitive impairment previously. Therefore, family members providing care may be unsure of what may be perpetuating their loved one’s functional problems or how they can help, even if they are willing and able to step into a direct caregiving role. Accordingly, timely provision of psychoeducation resources may help to bridge the gap for caregivers, who may then be better equipped to identify areas of cognitive difficulty, track any changes over time, share concerns from a real-world environment with clinical teams, and modify their behavior or the environment to better support their loved one.

Research examining the impact of cognition-specific education resources for families supporting people with psychosis is limited; however, caregivers involved in cognitive intervention for psychosis have previously reported that learning about cognitive impairment is “*extremely helpful*” (ie, 93%).^31^ While novel, these findings are also unsurprising given that educational interventions for families, such as those focused on psychosis symptoms, treatments, and skill development, have been shown to reduce caregiver burden and distress.^32–35^ This is likely via their positive influence on factors such as illness-related knowledge, self-confidence, social connection, and self-appraisal about caregiving.^34^ Additionally, caregivers participating in family-led cognitive adaption treatments have previously expressed a high level of interest in learning about simple cognitive compensation strategies or environmental changes that can be easily applied in daily life to support their loved one (eg, alarms, signs, checklists, routines, repetition).^36^ There is also promising evidence that the use of manualized cognitive compensation materials, designed specifically for families, may produce moderate-to-large improvements in community functioning among consumers and large declines in caregiver burden.^36^ Therefore, information provision about cognitive problems and concrete strategies may be a reasonable and low-cost *minimum* standard for caregivers supporting a person with psychosis. Compensatory strategies have a strong evidence base in psychosis^5^ and are viewed favorably by both mental health clinicians and consumers in early psychosis settings,^6,37^ which may facilitate transfer of information within members of the support network.

There are a range of existing cognition-specific educational resources for families caring for people with psychosis. For example, Medalia and Revheim^38^ have published the second edition of their free, web-accessible English and Spanish language handbook for families and friends. This provides information about cognitive impairment in psychiatric illnesses, their impact on functional recovery, the factors that make them better or worse, cognitive remediation, and the specific strategies families can use to support cognitive health. Additionally, Cellard and colleagues^39^ have recently published a suite of cognition resources in French and English, including toolkits for consumers, families and professionals, that offer accessible information about the brain and cognitive impairment as well as practical solutions for daily management. There are also an increasing number of online resources about cognitive health for families, including web-based articles, manuals, pamphlets, videos, and audio clips (eg, www.teachrecovery.com; https://patient.boehringer-ingelheim.com/disease/cns/psychiatry/schizophrenia-education/cognitive-impairment#symptoms; www.webmd.com/schizophrenia/toc-schizophrenia-caregiving).

However, by and large, most families do not know that these resources exist or where to find them. Clinicians do not regularly discuss cognitive functioning or share these cognition resources with consumers or their families due to their lack of perceived knowledge about cognitive health.^37^ Further, the impact of cognitive health promotion resources on caregiving-related factors including knowledge, confidence, coping ability, or psychological well-being has not been systematically evaluated. Psychoeducation resources and interventions focused on increasing knowledge of cognitive symptoms and compensatory strategies are intended to help caregivers supporting people with psychosis, but the precise impact is unknown. This research could potentially enhance the use of cognitive health psychoeducation materials as part of evidence-based guidelines for managing cognitive impairment.^36^

## Caregivers Could Have an Active Role in Cognitive Treatment Compliance or Delivery

Beyond psychoeducation and simple management strategies, there may also be a role for caregivers with adequate capacity to be active participants in cognitive-enhancing or compensatory treatments for people with psychosis in some settings. Many behavioral treatments aimed at reducing the impact of cognitive impairment on everyday functioning, such as cognitive remediation or compensation, involve active skill development and repetitive practice to improve cognitive abilities and/or use compensatory strategies to work around cognitive problems.^40^ One fundamental goal of these treatments is generalization to community settings, which may be more likely to when new skills and strategies are practiced within real-world settings (eg, the home environment). While clinicians are encouraged to support consumer participation in real-world transfer activities, there is an inevitable expectation that consumers complete homework tasks and are eventually able to demonstrate their cognitive gains in the community (eg, identify and effectively implement a memory strategy in an appropriate situation).^41^ Thus, if offered guidance, caregivers may have a critical supporting role in providing prompts to practice newly acquired skills or modify the environment to optimize treatment success.^36^ A caregiver’s knowledge of a consumer’s strengths, motivations, and preferences could be utilized to maximize success. This may be a logical progression for caregivers who have already received psychoeducation about cognitive impairment in psychosis and may be more equipped to implement basic cognitive strategies to support treatment participation (eg, helping a consumer to use or check memory aides or establish plans for using new strategies routinely; see Medalia & Revheim^38^). Existing research has shown that family-directed cognitive adaption treatments can improve consumer functioning and reduce family burden,^31^ thus highlighting the positive impact of some care-led interventions on consumer and carer outcomes.

In future, caregivers may also play an active role in facilitating treatments directly targeting cognitive impairment in psychosis that have traditionally been reserved for trained therapists, such as cognitive remediation. In the last decade, there has been growing interest in home-based delivery of cognitive remediation, with and without caregiver involvement. This may be because remote forms of treatment are acceptable by consumers and their families and may also mitigate some of the implementation problems of traditional forms of treatment (eg, time constraints, limited geographical access, poor digital literacy, skills, and confidence).^42–45^ There is some evidence that home-based, caregiver-assisted cognitive remediation may produce equivalent levels of cognitive improvement as treatments offered in clinic-based settings.^46^ Nevertheless, more research on the feasibility and efficacy of this treatment and the preferences of caregivers is needed before clear recommendations about their involvement can be made. Some concerns associated with caregiver-led cognitive treatments may include elevated rates of attrition; the time, energy, and responsibility required from an already under-resourced group of people; the mental, physical, and economic cost; and the potential for interpersonal conflict (eg, blurred boundaries between their role as a family member and therapist). There is also a risk that treatment becomes less client-centered if focused on the cognitive treatment needs viewed through the lens of the family, which may or may not overlap with the consumer’s preferences. Accordingly, it is possible that an unintended consequence of active caregiver participation is *increased* caregiver burden or blurred boundaries between family and therapeutic roles, which may negatively affect treatment outcomes. This requires further investigation.

## Moving Forward in Supporting Caregivers: Challenges and Opportunities

There is a critical lack of guidance in how best to support or involve caregivers in cognitive health promotion, despite the knowledge that caregivers struggle with cognitive impairments of loved ones with psychosis and that these impairments contribute to aspects of functional disability and exacerbate caregiver burden. Accordingly, there is a need to better understand the cognitive treatment needs and perspectives of caregivers who are providing support to loved ones with psychosis and how the cognitive symptoms uniquely impact caregiver capacity and distress. There is also a need to work collaboratively with consumers and their families to develop, personalizes, and implement cognitive supports and better understand the enablers and barriers to family involvement in this space, which may inform better cognitive and functional treatments.^7^

There is also a need to appreciate the challenges associated with recommendations for greater family involvement in the cognition treatment landscape. Family participation is recommended in clinical practice guidelines for psychotic illnesses, but it is notoriously difficult to implement in the real world. For example, previous research has shown that family interventions are seldom offered to or received by adults with psychotic disorders in public mental health services (ie, < 2%).^47^ Common barriers to implementing family interventions are related to limited clinical capability (eg, lack of training and supervision, large caseloads, competing priorities) as well as low family/consumer interest and readiness, inability to access support, and difficulty meeting individual family needs/preferences (eg, timing, length, intensity, format and content).^47,48^ Perceived outcomes may also differ according to stakeholders groups, which may become a significant barrier if not assertively addressed at the outset. For example, while breaches of privacy are often a shared concern, families may worry about their participation intruding upon the consumer–clinician relationship, while consumers may be concerned about family involvement diminishing their autonomy.^49,50^

Many of these barriers are similar to those described in the cognitive treatment for psychosis literature. These include: minimal opportunities for workforce training and supervision; competing clinical priorities or underappreciation of cognition as priority treatment target; consumer-related factors (eg, motivation, cognitive awareness); intervention-related factors (eg, treatment length, intensity), and inconsistent organizational support, endorsement or infrastructure (eg, lack of resources, fluctuating commitment to education and quality assurance).^51–53^ Thus, there are reasons to anticipate that assisting caregivers with understanding and actively addressing the cognitive health needs of people with psychotic disorders will be challenging, even if the families themselves are willing and able to assist and have the support to do so from their loved one. These limitations will require careful attention when working with families to address their cognition-specific caregiving needs (eg, acknowledgment of time constraints affecting a clinician’s capacity to offer consistent support). Future research exploring the impact of cognition resources for families or their involvement in cognitive treatment must do so while gathering evidence for implementation (eg, individual, intervention, financial, and organizational factors). This must take into consideration the perspectives of multiple stakeholders, including those less inclined to support the cognitive needs of their loved one, to facilitate greater understanding, uptake, and sustainability.^54^

In the absence of formal guidelines, we offer some recommendations for clinical teams working with people with psychosis who have a caregiver that they want to be involved in cognitive treatment and who has the willingness and capacity to participate at some level (see Table 1). Some of the practical resources discussed in this article, such as those describing compensatory strategies for daily life, may also be accessible for nonfamily support persons/networks (eg, support workers, housing workers, coaches). These recommendations draw upon the existing cognitive literature and seek to increase caregiver capacity and capability. They are based on the experience of the authorship team who have varying experiences in the mental health sector, including senior academics, clinical neuropsychologists, implementation scientists, and those with lived experience as a mental health consumer or as a family member of a loved one with a psychotic disorder. This is a call for clinical and research action.

**Table 1.** Call to Action: Practical Recommendations for Clinical Teams Supporting Family Members Who Provide Care to Loved Ones With a Psychotic Disorder

**(1) Recognize and validate the difficulties faced by caregivers** Ask the caregiver what they know about cognitive problems in psychotic disorders, what they want to know, how they think the clinical team can help with cognition, and what supports they may need to help them manage. Use this information to frame ongoing consultations Recognize that cognitive impairment as a prominent feature for most people with psychosis Normalize the shock or distress that impairment can cause them and their family. Identify the ways in which the caregiving relationship may have changed or could change in the future (eg, consumer needing more support with daily organization or advocacy) Appreciate that some caregivers may have had poor experiences with mental health services or may have received limited supports previously, which may cause psychological reactions that also have to be managed (eg, distress or frustration)
**(2) Offer psychoeducation about cognitive impairment in psychosis** Provide timely education about cognitive difficulties in psychosis to caregivers. This may include their relationship with clinical symptoms, trajectory over time, or functional impact on areas that make sense to the caregiver (eg, impact of memory problems on work). Clinicians may need to upskill in this area to fill competency gaps with professional development, which may include online resources, short courses, and supervision (www.medscape.org/viewarticle/1000445; www.orygen.org.au/Training/Resources/Cognition) Consider the caregiver’s readiness and capacity to understand the information as well as the consumers stage of illness (eg, providing basic leaflets about cognition during acute illness phases and lengthier resources, such as caregiver handbooks, during maintenance phases). Avoid clinical jargon and draw upon concrete analogies or metaphors that help family members to understand cognitive functioning (eg, filing cabinet analogy for memory encoding and retrieval) If possible, offer caregivers an opportunity to speak with members of the treating team (or attend family meetings) about their loved one’s cognitive challenges
**(3) Connect caregivers with meaningful support networks** Cognitive symptoms contribute to caregiver burden. Thus, if appropriate, provide caregivers with opportunities to make meaningful connections with “other people that get it,” such as carer peers with lived experience. Peer support may result in resource sharing (eg, tips). Consider the ways that caregivers can also support themselves (eg, professionals that can provide them with psychological care) or who else in the support network can offer support. This may help caregivers to remain hopeful and ease possible signs or symptoms burnout
**(4) Work with the caregiver to gather or disseminate information about cognitive health** Recognize that caregivers are uniquely placed in the support network and may have in-depth knowledge about their loved one’s strengths and any new, existing, or anticipated areas of weakness. In consultation with the consumer, speak with the caregiver and draw upon their knowledge and identify areas that require additional support, negotiation or planning (eg, memory problems are being observed by the carer and are impacting their loved one’s retention of daily tasks, which may require careful consideration as part of their functional recovery). If a formal cognitive assessment or screening will be conducted, ask the consumer whether it is OK to share the results with their family or if they would like to consider involving them in the feedback process. Consumers may not spontaneously suggest this in practice. Seek support on the ways to communicate information about cognitive functioning with their loved one (eg, preferences for written materials)
**(5) Actively involve caregivers in cognitive health promotion and recovery (if possible)** Support caregivers to learn about the types of evidence-based treatments for cognitive impairment that may be accessible to them and what could be implementable in their context (eg, cognitive compensation, exercise). This may include how existing treatments may help or hinder their abilities (eg, sedation associated with medication). Specialist cognitive enhancing treatments, such as cognitive remediation, may not be available in most settings; however, if it is, advocating for family involvement in specific aspects could be beneficial if clinically indicated. There is no framework for how this should occur so open discussions about the needs of all parties and the pros and cons of participating should be discussed (eg, if cognitive remediation is available and is partly completed off-site, could it be helpful for family members to assist consumers with homework or monitor real-world gains or barriers to discuss these later with clinical teams?). Encourage caregivers to utilize simple cognitive management strategies to support their loved one’s independence in daily life and/or be involved in treatment when they are willing and able to provide this support (eg, translation of memory strategies from the clinic to the home, prompting homework completion). Direct them to accessible information found in reputable webpages, physical handbooks, pamphlets, videos or podcasts, may be a core focus in these discussions (see above). The process of supporting caregivers to be actively involved in treatment should be open and collaborative, balance the needs of the caregiver and consumer, and consider the possible impacts on the carer–consumer relationship (eg, consumer frustration due to behaviors that are paternalistic or overbearing). Encourage consumers to develop advance care statements about how they may want (or not want) to receive cognitive support from caregivers when they are unwell (eg, attendance in medical reviews, support with appointments)

It must be stated, however, that these suggestions are only a starting point for increasing caregiver involvement in cognitive health promotion in psychotic disorders, with a focus on increasing clinician and caregiver knowledge and capability. Research in this area is in its infancy and, accordingly, it is difficult to provide comprehensive evidence-based recommendations that are likely feasible in all implementation contexts. Despite this, we would argue that families and clinicians may still benefit from attaining practical knowledge about cognition, even in the absence of established cognitive treatment services (eg, clinicians offering cognitive remediation). For example, over half of front-line clinicians from one coordinated specialty care service for early psychosis in New York, NY (*N* = 53), indicated that a lack of knowledge about cognition is one of the biggest barriers to discussing cognitive health with clients with psychotic disorders (52%). However, approximately 9 in 10 (88%) reported that they would be “*very likely*” to integrate cognitive health practices into their work if they were given the tools and resources.^37^ Some of the most appealing methods of intervention for clinicians were manulized compensatory skills training (90%), working with family/support people (84%), and providing psychoeducation about cognitive impairment (76%).^37^ These options were preferred to cognitive training and referring the client out to another service.

We hope that some of these suggestions may be a focus of future consumer-oriented research studies seeking to understand the best ways of supporting willing and able families with understanding and addressing the cognitive problems in psychotic disorders. These initiatives are urgently needed and will be essential for moving clinical practice and research in this field forward.

## References

[1] Galletly C, Castle D, Dark F, et al Royal Australian and New Zealand College of Psychiatrists clinical practice guidelines for the management of schizophrenia and related disorders. Aust N Z J Psychiatry. 2016;50:410–472. 27106681 10.1177/0004867416641195

[2] Keepers GA, Fochtmann LJ, Anzia JM, et al The American Psychiatric Association practice guideline for the treatment of patients with schizophrenia. Am J Psychiatry. 2020;177:868–872. 32867516 10.1176/appi.ajp.2020.177901

[3] Vita A, Gaebel W, Mucci A, et al European Psychiatric Association guidance on treatment of cognitive impairment in schizophrenia. Eur Psychiatry: J Assoc Eur Psychiatr. 2022;65:e57. 10.1192/j.eurpsy.2022.2315 PMC9532218 36059103

[4] Vita A, Barlati S, Ceraso A, et al Effectiveness, core elements, and moderators of response of cognitive remediation for schizophrenia: a systematic review and meta-analysis of randomized clinical trials. JAMA Psychiatry. 2021;78:848–858. 33877289 10.1001/jamapsychiatry.2021.0620 PMC8058696

[5] Allott K, van-der-EL K, Bryce S, et al Compensatory interventions for cognitive impairments in psychosis: a systematic review and meta-analysis. Schizophr Bull. 2020;46:869–883. 32052837 10.1093/schbul/sbz134 PMC7345816

[6] Bryce S, Cheng N, Dalton A, et al Cognitive health treatment priorities and preferences among young people with mental illness: your mind, your choice survey. Early Intervent Psychiatry. 2024;18:94–101. 10.1111/eip.13436 37198726

[7] Gonzales L, Jones N. Service user representation in qualitative research on cognitive health and related interventions for psychosis: a scoping review. Schizophr Bull. 2024;50:1006–1016. 38525590 10.1093/schbul/sbae035 PMC11349025

[8] Citrome L, Belcher E, Stacy S, Suett M, Mychaskiw M, Salinas GD. Perceived burdens and educational needs of caregivers of people with schizophrenia: results of a national survey study. Patient Preference Adherence. 2022;16:159–168. 35087268 10.2147/PPA.S326290 PMC8789274

[9] Doyle R, Turner N, Fanning F, et al First-episode psychosis and disengagement from treatment: a systematic review. Psychiatr Serv (Washington, D.C.). 2014;65:603–611. 10.1176/appi.ps.201200570 24535333

[10] Kuhnigk O, Slawik L, Meyer J, Naber D, Reimer J. Valuation and attainment of treatment goals in schizophrenia: perspectives of patients, relatives, physicians, and payers. J Psychiatr Pract. 2012;18:321–328. 22995959 10.1097/01.pra.0000419816.75752.65

[11] Moritz S, Berna F, Jaeger S, Westermann S, Nagel M. The customer is always right? Subjective target symptoms and treatment preferences in patients with psychosis. Eur Arch Psychiatry Clin Neurosci. 2017;267:335–339. 27194554 10.1007/s00406-016-0694-5

[12] Stainton A, Cheng N, Bryce S, et al Cognition is a treatment priority for young people with psychosis: findings from the your mind, your choice survey. Schizophr Res. 2023;262:30–31. 37922840 10.1016/j.schres.2023.10.019

[13] Harvey C, Killackey E, Groves A, Herrman H. A place to live: housing needs for people with psychotic disorders identified in the second Australian national survey of psychosis. Aust N Z J Psychiatry. 2012;46:840–850. 22619384 10.1177/0004867412449301

[14] Tsai J, Stroup TS, Rosenheck RA. Housing arrangements among a national sample of adults with chronic schizophrenia living in the United States: a descriptive study. J Community Psychol. 2011;39:76–88.

[15] Onwumere J, Sirykaite S, Schulz J, et al Understanding the experience of “burnout” in first-episode psychosis carers. Compr Psychiatry. 2018;83:19–24. 29505884 10.1016/j.comppsych.2018.02.003

[16] Bryce S, Cheng N, Stainton A, et al Participation preferences in cognitive treatments among youth with mental illness: findings from the your mind, your choice survey. Early Intervent Psychiatry. 2024;19:e13615. 10.1111/eip.13615 39358808

[17] Cohen AN, Drapalski AL, Glynn SM, Medoff D, Fang LJ, Dixon LB. Preferences for family involvement in care among consumers with serious mental illness. Psychiatr Serv (Washington, D.C.). 2013;64:257–263. 10.1176/appi.ps.201200176 23242515

[18] Jones N, Basaraba C, Piscitelli S, et al Clients’ preferences for family involvement and subsequent family contact patterns within OnTrackNY Early psychosis services. Psychiatr Serv (Washington, D.C.). 2021;72:399–407. 10.1176/appi.ps.202000241 PMC10281455 33530730

[19] Brain C, Kymes S, DiBenedetti DB, Brevig T, Velligan DI. Experiences, attitudes, and perceptions of caregivers of individuals with treatment-resistant schizophrenia: a qualitative study. BMC Psychiatry 2018;18:253. 30103719 10.1186/s12888-018-1833-5 PMC6090592

[20] Velligan DI, Brain C, Bouérat Duvold L, Agid O. Caregiver burdens associated with treatment-resistant schizophrenia: a quantitative caregiver survey of experiences, attitudes, and perceptions. Front Psychiatry. 2019;10:584. 31551821 10.3389/fpsyt.2019.00584 PMC6743609

[21] Reuteman-Fowler C, Tulliez S, Ridley Phd Cpsychol M, Rudell K, Girardi A, Correll C, Keefe R, Goldring A. Objective burden among primary and secondary caregivers from cognitive impairment symptoms of patients with schizophrenia; 2024.

[22] Cruz E, Paré MA, Stan C, Voth J, Ward L, Taboun M. Caring for the caregiver: an exploration of the experiences of caregivers of adults with mental illness. SSM—Qualitat Res Health. 2024;5:100406.

[23] Sin J, Elkes J, Batchelor R, et al Mental health and caregiving experiences of family carers supporting people with psychosis. Epidemiol Psychiatr Sci. 2021;30:e3. 33416043 10.1017/S2045796020001067 PMC7116786

[24] Lerner D, Chang H, Rogers WH, Benson C, Lyson MC, Dixon LB. Psychological distress among caregivers of individuals with a diagnosis of schizophrenia or schizoaffective disorder. Psychiatr Serv (Washington, D.C.). 2018;69:169–178. 10.1176/appi.ps.201600422 28967321

[25] Hegde A, Chakrabarti S, Grover S. Caregiver distress in schizophrenia and mood disorders: the role of illness-related stressors and caregiver-related factors. Nord J Psychiatry. 2019;73:64–72. 30638102 10.1080/08039488.2018.1561945

[26] Karambelas GJ, Allott KA, Byrne LK, et al A comparison of challenging and positive caregiving experiences for caregivers of individuals with schizophrenia spectrum and bipolar disorders. J Affect Disord Rep. 2024;18:100840.

[27] Allott K, Wood S, Nelson B, et al Cognitive Impairment in Psychosis: Landscape Report; 2021.

[28] Cheng SC, Backonja U, Buck B, Monroe-DeVita M, Walsh E. Facilitating pathways to care: a qualitative study of the self-reported needs and coping skills of caregivers of young adults diagnosed with early psychosis. J Psychiatr Ment Health Nurs. 2020;27:368–379. 31930633 10.1111/jpm.12591

[29] Petrova I, Pipere A. Caregivers’ experience of caring for family members with complex health needs in Latvia. Health Promot Int. 2024;39:daae070. 38934478 10.1093/heapro/daae070

[30] Wright AL, Phillips LJ, Bryce S, Morey-Nase C, Allott K. Subjective experiences of cognitive functioning in early psychosis: a qualitative study. Psychosis. 2019;11:63–74.

[31] Friedman-Yakoobian MS, Mueser KT, Giuliano AJ, Goff D, Seidman LJ. Family-directed cognitive adaptation pilot: teaching cognitive adaptation to families of individuals with schizophrenia. Am J Psychiatr Rehabil. 2016;19:62–74. 29167630 10.1080/15487768.2015.1125401 PMC5695924

[32] Riley G, Gregory N, Bellinger J, Davies N, Mabbott G, Sabourin R. Carer’s education groups for relatives with a first episode of psychosis: an evaluation of an eight-week education group. Early Intervent Psychiatry. 2011;5:57–63. 10.1111/j.1751-7893.2010.00195.x 21272276

[33] Claxton M, Onwumere J, Fornells-Ambrojo M. Do family interventions improve outcomes in early psychosis? A systematic review and meta-analysis. Front Psychol. 2017;8:371. 28396643 10.3389/fpsyg.2017.00371 PMC5366348

[34] Sin J, Gillard S, Spain D, Cornelius V, Chen T, Henderson C. Effectiveness of psychoeducational interventions for family carers of people with psychosis: a systematic review and meta-analysis. Clin Psychol Rev. 2017;56:13–24. 28578249 10.1016/j.cpr.2017.05.002

[35] Tessier A, Roger K, Gregoire A, Desnavailles P, Misdrahi D. Family psychoeducation to improve outcome in caregivers and patients with schizophrenia: a randomized clinical trial. Front Psychiatry. 2023;14:1171661. 37426102 10.3389/fpsyt.2023.1171661 PMC10326382

[36] Kidd SA, Kerman N, Ernest D, et al A pilot study of a family cognitive adaptation training guide for individuals with schizophrenia. Psychiatr Rehabil J. 2018;41:109–117. 27547853 10.1037/prj0000204

[37] Saperstein AM, Medalia A, Bello I, Dixon LB. Addressing cognitive health in coordinated specialty care for early psychosis: real-world perspectives. Early Intervent Psychiatry 2021;15:374–379. 10.1111/eip.12966 PMC8237375 32307919

[38] Medalia A, Revheim N. Dealing With Cognitive Dysfunction Associated With Psychiatric Disabilities. A Handbook for Families and Friends of Individuals With Psychiatric Disorders. New York: The New York State Office of Mental Health Family Liaison Bureau; 2002.

[39] Cellard C, East-Richard C, Racine E, et al Brain: toolkit for professionals as well as family and friends of people with neuropsychological difficulties. Clin Booklet. 2024. https://www.cerveau.psy.ulaval.ca/

[40] Bowie CR, Bell MD, Fiszdon JM, et al Cognitive remediation for schizophrenia: an expert working group white paper on core techniques. Schizophr Res. 2020;215:49–53. 31699627 10.1016/j.schres.2019.10.047

[41] Best MW, Milanovic M, Tran T, et al Motivation and engagement during cognitive training for schizophrenia spectrum disorders. Schizophr Res: Cogn. 2019;19:100151. 31828022 10.1016/j.scog.2019.100151 PMC6889769

[42] Parri LA, Barret K, Hill R, et al Evaluating the acceptability of remote cognitive remediation from the perspective of psychosis service users. Behav Cogn Psychother. 2024;52:495–507. 38347728 10.1017/S1352465824000109

[43] Cella M, Parri L, Wang K, et al Evaluating remote delivery of cognitive remediation in people with psychosis. Schizophr Res. 2024;267:367–372. 38631111 10.1016/j.schres.2024.04.001

[44] Jagtap S, Romanowska S, Leibovitz T, Onno KA, Burhan AM, Best MW. Can cognitive remediation therapy be delivered remotely? A review examining feasibility and acceptability of remote interventions. Schizophr Res: Cogn. 2022;28:100238. 35242607 10.1016/j.scog.2022.100238 PMC8861417

[45] Spanakis P, Wadman R, Walker L, et al Measuring the digital divide among people with severe mental ill health using the essential digital skills framework. Perspect Public Health. 2024;144:21–30. 35929589 10.1177/17579139221106399 PMC10757390

[46] Kumar D, Ashwini K, Hegde S, et al Caregiver assisted home-based cognitive remediation for individuals diagnosed with schizophrenia: a pilot study. Asian J Psychiatry. 2019;42:87–93. 10.1016/j.ajp.2019.03.010 PMC7613146 30981943

[47] Haddock G, Eisner E, Boone C, Davies G, Coogan C, Barrowclough C. An investigation of the implementation of NICE-recommended CBT interventions for people with schizophrenia. J Mental Health (Abingdon, England). 2014;23:162–165. 10.3109/09638237.2013.869571 24433132

[48] Selick A, Durbin J, Vu N, O’Connor K, Volpe T, Lin E. Barriers and facilitators to implementing family support and education in Early Psychosis Intervention programmes: a systematic review. Early Intervent Psychiatry. 2017;11:365–374. 10.1111/eip.12400 28418227

[49] Landeweer E, Molewijk B, Hem MH, Pedersen R. Worlds apart? A scoping review addressing different stakeholder perspectives on barriers to family involvement in the care for persons with severe mental illness. BMC Health Serv Res. 2017;17:349. 28506296 10.1186/s12913-017-2213-4 PMC5433083

[50] Tham SS, Solomon P. Family involvement in routine services for individuals with severe mental illness: scoping review of barriers and strategies. Psychiatr Serv (Washington, DC). 2024;75:1009–1030. 10.1176/appi.ps.20230452 38938096

[51] Altman RAE, Reser M, Tan EJ, Rossell SL. Cognitive remediation for schizophrenia: clinician perspectives on implementation barriers and facilitators. Rehabil Psychol. 2024;69:171–183. 38512182 10.1037/rep0000552

[52] Lammas F, Phillips A, Dopson S, Joyce E, Csipke E, Wykes T. The organisational climate of NHS Early Intervention Services (EIS) for psychosis: a qualitative analysis. BMC Health Serv Res. 2022;22:509. 35428229 10.1186/s12913-022-07790-0 PMC9013142

[53] Dark FL, Amado I, Erlich MD, Ikezawa S. International experience of implementing cognitive remediation for people with psychotic disorders. Schizophr Bull. 2024;50:1017–1027. 38758086 10.1093/schbul/sbae071 PMC11349011

[54] Zbukvic I, Bryce S, Moullin J, Allott K. The use of implementation science to close the research-to-treatment gap for cognitive impairment in psychosis. Aust N Z J Psychiatry. 2023;57:1308–1315. 36964703 10.1177/00048674231160987 PMC10517591

